# A review on the application of deep learning for CT reconstruction, bone segmentation and surgical planning in oral and maxillofacial surgery

**DOI:** 10.1259/dmfr.20210437

**Published:** 2022-05-13

**Authors:** Jordi Minnema, Anne Ernst, Maureen van Eijnatten, Ruben Pauwels, Tymour Forouzanfar, Kees Joost Batenburg, Jan Wolff

**Affiliations:** 1 Department of Oral and Maxillofacial Surgery/Pathology, Amsterdam UMC and Academic Centre for Dentistry Amsterdam (ACTA), Vrije Universiteit Amsterdam, 3D Innovationlab, Amsterdam Movement Sciences, Amsterdam, The Netherlands; 2 Institute for Medical Systems Biology, University Hospital Hamburg-Eppendorf, Hamburg, Germany; 3 Computational imaging group, Centrum Wiskunde & Informatica (CWI), Amsterdam, The Netherlands; 4 Department of Biomedical Engineering, Eindhoven University of Technology, Medical Image Analysis Group, Eindhoven, The Netherlands; 5 Aarhus Institute of Advanced Studies, Aarhus University, Aarhus, Denmark; 6 Leiden Institute of Advanced Computer Science (LIACS), Leiden University, Leiden, The Netherlands; 7 Department of Dentistry and Oral Health, Aarhus University, Vennelyst Boulevard, Aarhus, Denmark

**Keywords:** computer-assisted surgery, neural networks, CT image reconstruction, bone segmentation, surgical planning

## Abstract

Computer-assisted surgery (CAS) allows clinicians to personalize treatments and surgical interventions and has therefore become an increasingly popular treatment modality in maxillofacial surgery. The current maxillofacial CAS consists of three main steps: (1) CT image reconstruction, (2) bone segmentation, and (3) surgical planning. However, each of these three steps can introduce errors that can heavily affect the treatment outcome. As a consequence, tedious and time-consuming manual post-processing is often necessary to ensure that each step is performed adequately. One way to overcome this issue is by developing and implementing neural networks (NNs) within the maxillofacial CAS workflow. These learning algorithms can be trained to perform specific tasks without the need for explicitly defined rules. In recent years, an extremely large number of novel NN approaches have been proposed for a wide variety of applications, which makes it a difficult task to keep up with all relevant developments. This study therefore aimed to summarize and review all relevant NN approaches applied for CT image reconstruction, bone segmentation, and surgical planning. After full text screening, 76 publications were identified: 32 focusing on CT image reconstruction, 33 focusing on bone segmentation and 11 focusing on surgical planning. Generally, convolutional NNs were most widely used in the identified studies, although the multilayer perceptron was most commonly applied in surgical planning tasks. Moreover, the drawbacks of current approaches and promising research avenues are discussed.

## Introduction

Spatial information embedded in medical three-dimensional (3D) images is being increasingly used to personalize treatments by means of computer-assisted surgery (CAS). This novel image-based treatment modality enables clinicians to perform patient-specific virtual operations, 3D-print personalized medical constructs and perform robot-guided surgery.^1^ Moreover, CAS offers the unique possibility to conduct a limitless amount of different surgical simulations (osteotomies, grafts, implants, etc.) prior to surgery in a stress-free environment,^1^ and to predict surgical outcomes with minimal risk to the patient. Furthermore, such virtual simulations have proven to be useful for patient communication and medical education.^2,3^ As a result, CAS is currently being employed in multiple surgical branches involving the musculoskeletal system. In particular, CAS has advanced in the area of maxillofacial surgery.^4^

**Figure 1.**
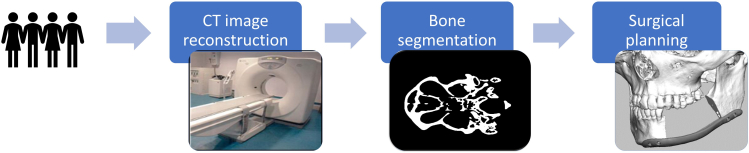
Schematic overview of the maxillofacial CAS workflow. CAS, computer-assisted surgery.

The current maxillofacial CAS workflow consists of different steps that are illustrated in Figure 1. The first step in the workflow is image acquisition. To date, numerous imaging modalities have become available on the market, including CT and MRI. CT imaging modalities are most commonly used to visualize bony structures due to their superior hard tissue contrast. CT scanners acquire X-ray projections of the patients’ anatomy from multiple angles. These projection data can be subsequently reconstructed into a 3D image using a wide variety of reconstruction methods. After the CT image acquisition step, image processing is necessary to convert the CT scans into a virtual 3D model in the standard tessellation language (STL) file format. This file format is supported by all FDA-approved medical software packages that are currently used for computer-aided design (CAD) and computer-aided manufacturing. The most important step in this CT-to-STL conversion is image segmentation (Figure 1), in which clinicians define and delineate anatomies of interest such as bone. In the final step of the CAS workflow, the acquired STL models are exported to dedicated medical CAD software packages and used for surgical planning by virtually designing patient-specific implants, surgical guides and radiotherapy boluses.^5^

Each of the aforementioned steps (*i.e.* CT image reconstruction, bone segmentation and surgical planning) is a potential source of errors which can lead to inaccuracies in the final STL models and impair the treatment outcome.^6^ For example, imaging noise, metallic structures and patient movements can heavily affect the CT image quality after reconstruction. For image segmentation, the segmentation technique can have a considerable effect on the accuracy of the resulting model.^6^ Furthermore, the surgical planning step currently relies on extensive domain expertise and manual software input, which often hampers its reproducibility.

One way to overcome these limitations is to employ neural networks (NNs) during the different steps of this maxillofacial CAS workflow. These learning algorithms are different from traditional computer methods in that they can be trained to find characteristic features and patterns in data, without the need for explicit rules specified by domain experts. The most common NN is the multilayer perceptron (MLP), which consists of an input layer, several hidden layers and an output layer. Each of these layers comprises several computational building blocks called neurons. Within an MLP, neurons are connected to neurons in subsequent layers. The output of each neuron is the product of its input with a learned set of weights plus a learned bias. Finally, a non-linear activation function is applied.^7^ It can be proven that MLPs can approximate any continuous function (universal approximation theorem,^8^ which gives them the ability to infer descriptive functions from data).

In the training phase, the weights and biases of a NN are learned from training data. During this training process, a large amount of input data is propagated through the NN to predict the values of the output layer. The goal of training is to minimize the difference between the NN prediction and the desired output by iteratively updating the weights and biases of the network. After optimizing these trainable parameters, NNs can be used to automatically perform specific tasks of the maxillofacial CAS workflow.

Recent advances in computational power and the development of novel NN algorithms have brought about a paradigm shift in the CAS workflow.^9^ A wide variety of advanced NN architectures have been successfully employed for various tasks such as image reconstruction and segmentation. For example, convolutional neural networks (CNNs) are especially useful for processing image data. These networks apply convolutions instead of a set of multiplications to compute the output of the individual layers. Another important type of neural networks is the recurrent neural network (RNN), which can handle temporal dynamic data. However, due to the rapidly increasing number of studies published in the field, maxillofacial surgeons and medical engineers have been facing the difficult task of keeping up with all developments. Therefore, this scoping review aims to provide an overview of the different types of NN approaches that have been used during the three main steps required in the CAS workflow, *i.e.* CT image reconstruction, bone segmentation and surgical planning. Furthermore, the secondary goal of this review paper is to identify the current bottlenecks and possible next research steps regarding the application of NNs in the maxillofacial CAS workflow.

## Methods and materials

Existing literature on the application of NNs in the maxillofacial CAS workflow was obtained using Pubmed, Embase, Scopus, Web of Science, and Google Scholar. An initial database was generated with the following search terms:

1. (CT OR CBCT OR computed tomography OR cone-beam computed tomography) AND (image reconstruction OR image processing OR image analysis OR image segmentation) AND (artificial intelligence OR deep learning OR neural network)
2. (bone OR bones OR bony) AND ((implant OR prosthesis OR virtual model) AND (design OR planning OR construct OR model)) AND (artificial intelligence OR deep learning OR neural network)

It must be noted that there was no specific focus on maxillofacial surgery when defining the search terms. The reason for this is that we believe that many of the techniques and methods used in other fields can also be of relevance to maxillofacial CAS. Choosing more generic search terms thus allowed us to identify a wider variety of literature relevant that was potentially relevant to maxillofacial CAS.

Publications were only included in the initial database if the search terms one or two were found in their title, abstract or keywords. After removing duplicates and adding literature found from references, a database of 6994 papers was acquired. The title and abstract of these publications were screened, resulting in 248 publications that were eligible for full-text reading. In order to assess whether papers were eligible for inclusion, the following inclusion and exclusion criteria were used:

- Described the development or implementation of a NN.
- Performed at least one of the three main steps required in the maxillofacial CAS workflow, *i.e.* CT image reconstruction, bone segmentation and surgical planning. Although surgical planning is an extremely broad field, this review specifically focused on the designing and optimization of implants and virtual models.
- Evaluated on medical data sets or artificial medical phantoms.

Inclusion criteria:

- Described the development or implementation of a NN.
- Performed at least one of the three main steps required in the maxillofacial CAS workflow, *i.e.* CT image reconstruction, bone segmentation and surgical planning. Although surgical planning is an extremely broad field, this review specifically focused on the designing and optimization of implants and virtual models.
- Evaluated on medical data sets or artificial medical phantoms.

Exclusion criteria:

- Used non-CT based imaging modalities.

**Figure 2.**
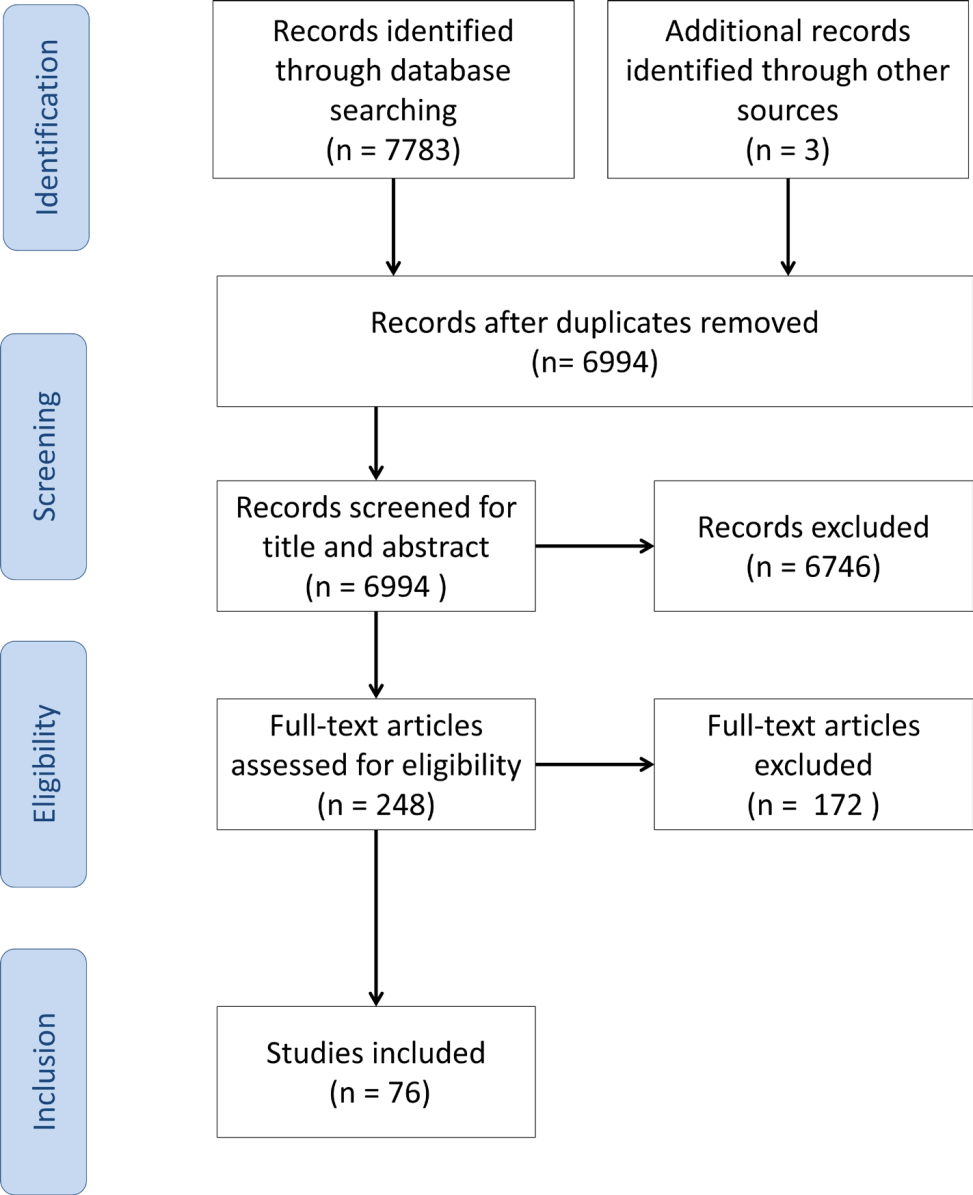
Overview of the study selection process of the present study.

The study selection process of the present study is shown in Figure 2.

## Results and discussion

**Figure 3.**
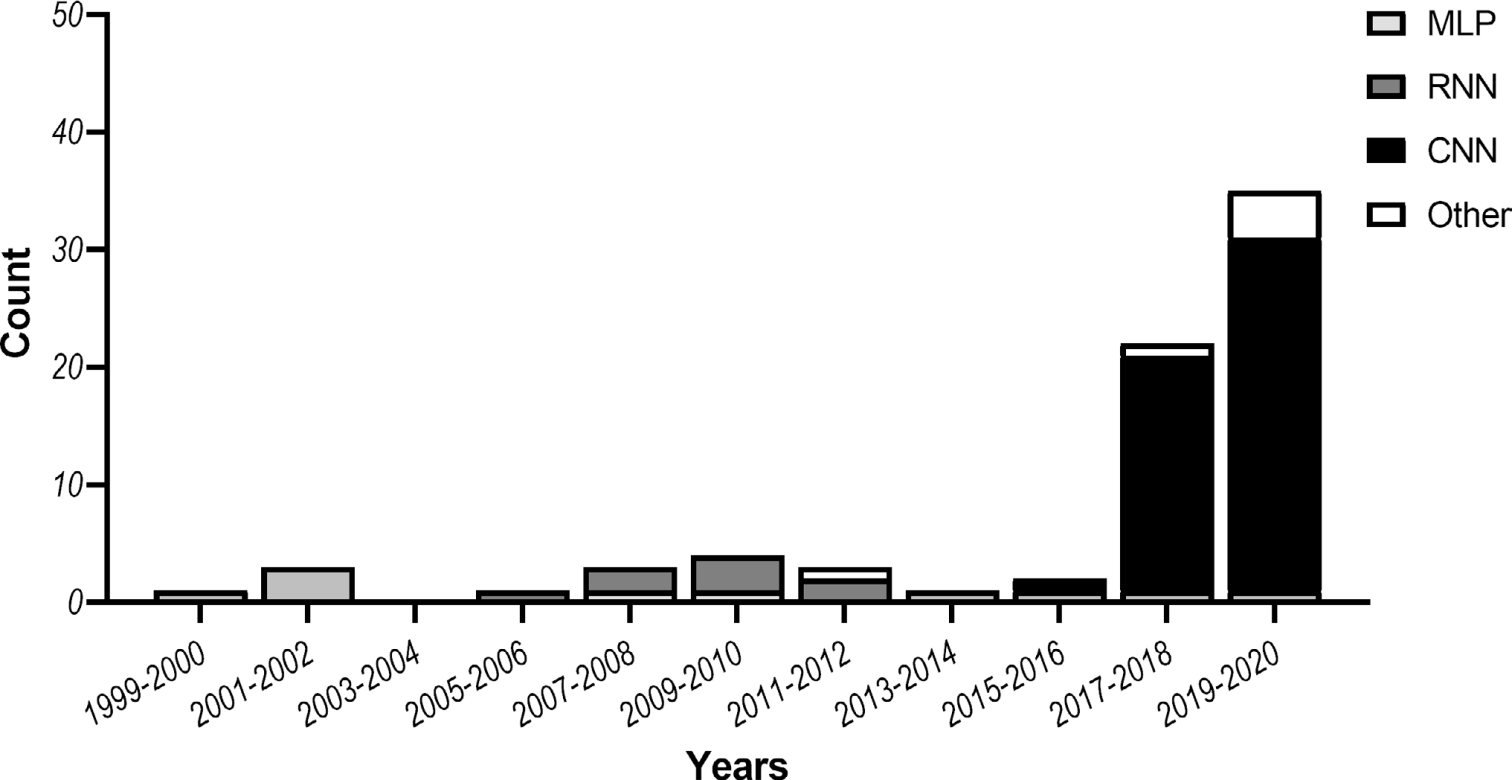
Most popular NN approaches in the maxillofacial CAS workflow over the past two decades. CAS, computer-assisted surgery; CNN, convolutional neural network; MLP, multilayer perceptron; NN, neural network; RNN, recurrent neural network.

This review aimed to identify NN architectures, training strategies and workflows that can potentially benefit CT image reconstruction, bone segmentation or surgical planning, since these steps are pivotal in the CAS workflow. In total, 76 studies were included in this review: 32 focusing on CT image reconstruction, 33 focusing on bone segmentation and 11 focusing on surgical planning. All studies are summarized in Table 1. In addition, Figure 3 shows the most popular NN approaches used in the reviewed studies.

**Table 1.** Overview of the studies included in this review

CAS step	Year	CT imaging modality	Anatomy	Neural network architecture	Authors
**CT image reconstruction**	2005	Fan-beam	Abdomen	Radial basis-function NN	Hu^10^
	2006	Parallel-beam	Shepp-Logan phantom	Radial basis-function NN	Guo^11^
	2008	Parallel-beam	Shepp-Logan phantom	RNN	Cierniak^12^
	2008	Parallel-beam	Shepp-Logan phantom	RNN	Cierniak^13^
	2009	Fan-beam	Shepp-Logan phantom	RNN	Cierniak^14^
	2010	Parallel-beam	Shepp-Logan phantom	RNN	Cierniak^15^
	2010	Parallel-beam	Shepp-Logan phantom	RNN	Cierniak^16^
	2011	Parallel-beam; Fan-beam	Shepp-Logan phantom	RNN	Cierniak^17^
	2012	Parallel-beam	Shepp-Logan phantom	RNN	Cierniak and Lorent^18^
	2016	Parallel-beam, Fan-beam	Chest	CNN	Würfl et al.^19^
	2017	Fan-beam	Shepp-Logan phantom, anthropomorphic phantom head	CNN	Adler and Öktem^20^
	2018	Parallel-beam, Fan-beam	Ellipse phantom, Shepp-Logan phantom, Human phantoms	CNN	Adler and Öktem^21^
	2018	Multidetector row	Abdomen	CNN	Chen et al.^22^
	2018	Multidetector row	Abdomen, Chest, Rat brain	CNN	Gupta et al.^23^
	2018	Multidetector row	Abdomen	CNN	Han et al.^24^
	2018	Fan-beam	Chest	CNN	Liang et al.^25^
	2018	Cone-beam	Abdomen	CNN	Würfl et al.^26^
	2019	Fan-beam	Skull	CNN	Dong et al.^27^
	2019	Parallel-beam	Chest	CNN	Fu and de Man^28^
	2019	Multidetector row	Torso	CNN	He et al.^29^
	2019	Multidetector row	Chest	CNN	Lee et al.^30^
	2019	Fan-beam	Skull	GAN	Li et al.^31^
	2019	Multidetector row	Chest	CNN	Shen et al.^32^
	2019	Fan-beam	Abdomen	CNN	Wu et al.^33^
	2019	–	Shepp-Logan phantom	CNN	Zhang and Zuo^34^
	2019	Cone-beam	Chest	CNN	Zhang et al.^35^
	2020	Fan-beam	Prostate	CNN	Chen et al.^36^
	2020	Translational CT	Chest	CNN	Wang et al.^37^
	2020	Parallel-beam	Chest	CNN	Baguer et al.^38^
	2020	Parallel-beam	Chest	CNN	Ma et al.^39^
	2020	Fan-beam; Cone-beam	Chest	CNN	Wang et al.^40^
	2020	Cone-beam	Breast	GAN	Xie et al.^41^
**Bone segmentation**	2002	Multidetector row	Chest, Skull	MLP	Zhang and Valentino^42^
	2008	Multidetector row	Phalanx	MLP	Gassman et al.^43^
	2013	-	Jaw, mouth, nose, eye, brain	MLP	Kuo et al.^44^
	2017	Multidetector row	Femur	CNN	Chen et al.^45^
	2017	–	Mandible, Spinal cord	CNN	Ibragimov and Xing^46^
	2017	Multidetector row	Femural head, bladder, intestine, colon	CNN	Males et al.^47^
	2018	Multidetector row	Whole body	CNN	Klein et al.^48^
	2018	Multidetector row	Vertebrae	CNN	Lessman et al.^49^
	2018	Multidetector row	Skull	CNN	Minnema et al.^50^
	2018	–	Vertebrae	Deep-belief network	Qadri et al.^51^
	2018	Multidetector row	Mandible	CNN	Yan et al.^52^
	2018	Multidetector row	Vertebrae	CNN	Zhou et al.^53^
	2019	–	Vertebrae	CNN	Dutta et al.^54^
	2019	–	Teeth	CNN	Gou et al.^55^
	2019	Multidetector row	Whole-body	CNN	Klein et al.^56^
	2019	Multidetector row	Orbital bones	CNN	Lee et al.^57^
	2019	Multidetector row	Vertebrae	CNN	Lessmann et al.^58^
	2019	Cone-beam	Mandible, teeth	CNN	Minnema et al.^4^
	2019	Multidetector row	Vertebrae	CNN	Rehman et al.^59^
	2019	Cone-beam	Skull	CNN	Torosdagli et al.^60^
	2019	–	Vertebrae	CNN	Vania et al.^61^
	2019	Multidetector row	Pelvic bones	CNN	Wang et al.^62^
	2019	Micro-CT	Teeth	CNN	Yazdani et al.^63^
	2020	Cone-beam	Teeth	CNN	Lee et al.^64^
	2020	–	Temporal bone	CNN	Li et al.^65^
	2020	–	Vertebrae	CNN	Yin et al.^66^
	2020	Multidetector row	Whole-body	CNN	Noguchi et al.^67^
	2020	Multidetector row	Vertebrae	CNN	Bae et al.^68^
	2020	Multidetector row	Cochleae	CNN	Heutink et al.^69^
	2020	Cone-beam	Skull	CNN	Zhang et al.^70^
	2020	–	Vertebrae	Cascaded CNN	Xia et al.^71^
	2020	Cone-beam	Teeth	CNN	Chen et al.^72^
	2020	Cone-beam	Teeth	CNN	Rao et al.^73^
					
**Surgical planning**	2000	–	Skull	MLP based on legendre polynomials	Hsu and Tseng^74^
	2001	–	Skull	MLP based on legendre polynomials	Hsu and Tseng^75^
	2001	Multidetector row	Radius	Bernstein Basis function network	Knopf and Al-Naji^76^
	2010	n.a.	Femur	MLP	Hambli^77^
	2012	n.a.	Femur	MLP	Campoli et al.^78^
	2012	n.a.	Bone microstructure	Meshing growing neural gas (MGNG)	Fischer and Holdstein^79^
	2016	n.a.	Femur	MLP	Chanda et al.^80^
	2018	n.a.	Dental implant	MLP	Roy et al.^81^
	2019	n.a.	Spinal implant	MLP	Biswas et al.^82^
	2020	–	Skull	GAN	Kodym et al.^83^
	2020	–	Mandible	GAN	Liang et al.^84^

- : not specified; CNN: convolutional neural network; GAN: generative adversarial network; MLP: multilayer perceptron;NN: neural network; RNN: recurrent neural network; n.a.: not applicable.

**Figure 4.**
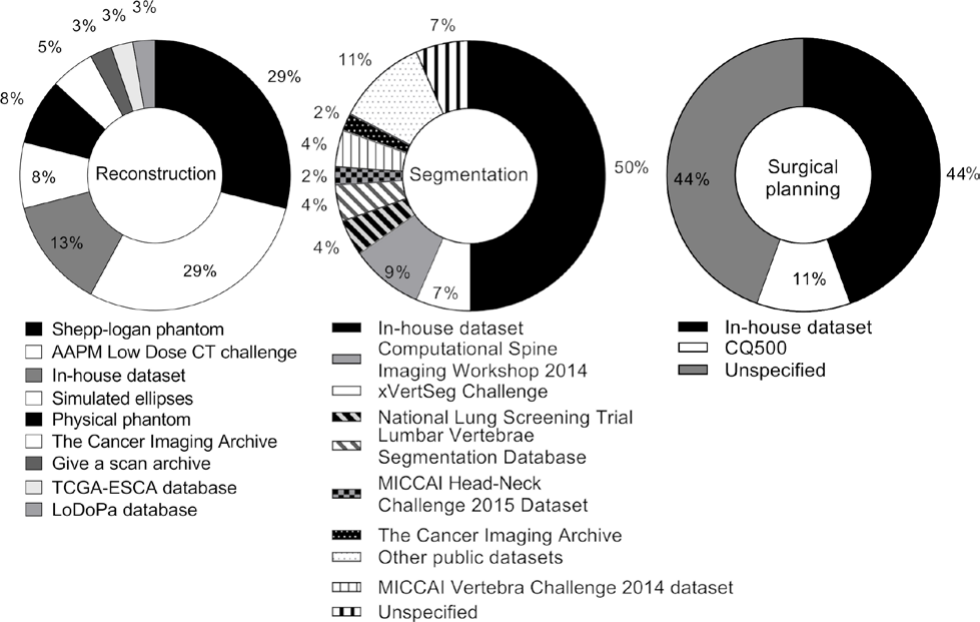
The data sets that were used to train and test the NN approaches in the reviewed studies. NN, neural network.

Of the 32 reviewed studies for CT image reconstruction, 19 studies used simulated CT data to train and test their NN approach, 15 studies used clinical CT data, and 2 studies used CT data of physical phantoms. In contrast, all 33 bone segmentation studies used clinical data sets to train and test the NN approaches. two surgical planning studies were performed based on clinical data sets in two studies, and the remaining nine studies were performed based on simulated data. Details of the data sets used to train the NN approaches are provided in Figure 4.

Training and testing of the NNs with clinical data was performed with a mean of 33 ± 42 CT volumes and 12 ± 16 CT volumes, respectively. The mean ratio between the amount of training and testing data was approximately 9:2. Furthermore, 13 of the 76 reviewed studies employed a leave-k-out testing strategy to improve validation of the NNs. In this cross-validation strategy, the available data are split into *k* folds, where one fold is alternately used as testing data, and the remaining folds serve as training data for the NN. This process is repeated *k* times such that all folds have been used for testing.

**Figure 5.**
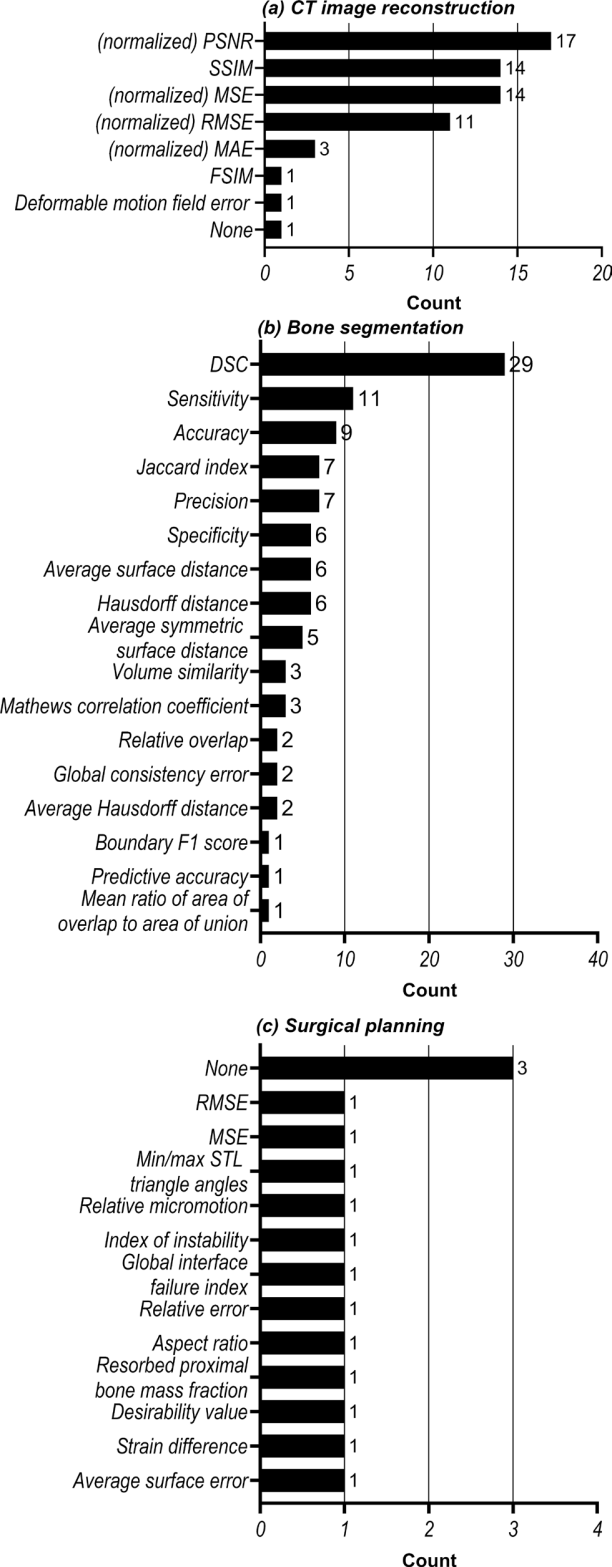
Analysis of the evaluation metrics used to quantify the performance of NN approaches in (**a**) CT image reconstruction, (**b**) bone segmentation and (**c**) surgical planning. DSC, dice similarity coefficient; RMSE, root meat-squared error; MSE, mean-squared error; PSNR, peak signal-to-noise ratio; SSIM, structural similarity index measure; STL, standard tessellation language.

Quantitative evaluation of the NNs’ performances for CT image reconstruction tasks was most commonly performed using the peak signal-to-noise ratio (PSNR) and the structural similarity index measure (SSIM) (Figure 5a). The bone segmentation NNs were most commonly evaluated using the dice similarity coefficient (DSC) (Figure 5b). No consistency was observed in the performance metrics used to evaluate the surgical planning step (Figure 5c). Moreover, 3 of the 11 surgical planning studies did not include quantitative performance evaluations.

In the following subsections, we elaborate on the NN approaches that have been employed in each of the three steps of maxillofacial CAS.

### Image reconstruction

**Figure 6.**
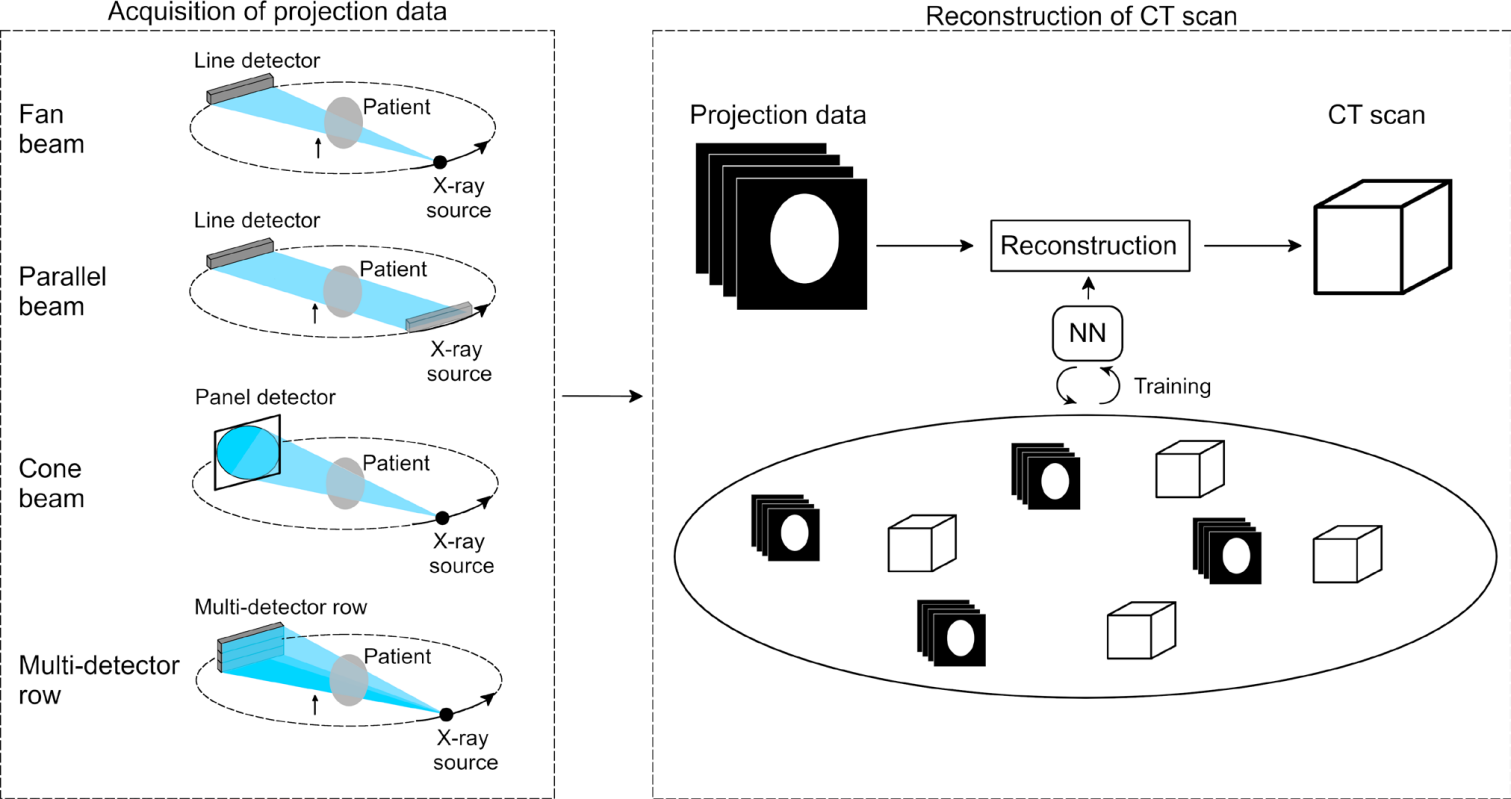
Schematic overview of CT image reconstruction. First, CT projection data are acquired with one of the four commonly used CT geometries (*i.e.* fan-beam, parallel-beam, cone-beam and multidetector row). The acquired projection data are used to reconstruct a CT scan. This reconstruction step can be improved by training a NN. NN, NN, neural network.

Historically, CT image reconstruction (Figure 6) has been a notoriously difficult task, which aims to compute the density of objects or anatomical structures based on the attenuation of X-rays. To date, two different reconstruction methods have been predominantly employed in clinical settings: filtered backprojection (FBP) and iterative reconstruction (IR). FBP is an analytical method in which measured projection data are uniformly distributed across the CT scan with an angle that corresponds to the acquisition of the projection data. A filter is subsequently applied to reduce blurring in the CT scan. By using projection data acquired at multiple angles with respect to the patient, a 3D CT scan can be reconstructed. IR approaches start similar to the FBP in that they use the measured projection data to reconstruct an initial CT scan. Based on this initial scan, a forward operation is performed to create artificial projection data. The artificial projection data are then compared to the measured projection data, which are used to update the initial CT scan. The forward operation and the scan update are repeated until the quality of the CT scan is satisfactory or for a fixed number of iterations. To date, a wide variety of different IR algorithms have been developed, including the algebraic reconstruction technique (ART), the simultaneous iterative reconstruction technique (SIRT) and model-based iterative reconstruction (MBIR).

Over the last decade, NNs have opened up a wealth of opportunities in the field of CT image reconstruction, offering CT images with higher quality than with FBP, while requiring shorter reconstruction times than current IR reconstruction approaches. One of the first efforts in developing NNs for medical CT image reconstruction can be traced back to 2005, when Hu et al proposed two different NN-based approaches.^10^ The first approach aimed to reconstruct 2D CT images using a Radial Basis Function NN (RBF-NN), which is a similar to the classical MLP, but uses a radial basis function as non-linear activation function. The input of this RBF-NN consisted of CT projection data, and the desired target consisted of previously reconstructed CT scans. In the second approach, the RBF-NN was employed to iteratively estimate the intensities of the voxels in the CT scan. More specifically, an IR scheme was employed in which the RBF-NN was trained to update the voxel values in the CT scans. Although both RBF-NN-based approaches were initially used to reconstruct small 32 × 32 images with only 8–16 projection angles, image sizes were increased to 128 × 128 in a later study by Guo et al.^11^

In a series of publications between 2008 and 2012,^12–18^ Cierniak et al developed multiple RNNs to iteratively reconstruct CT scans after using traditional backprojection to create an initial CT scan. The proposed RNNs were essentially used as learnable filters for the FBP reconstruction method, replacing the fixed filters in FBP.^17^ Cierniak demonstrated that the proposed RNNs improved CT image quality compared to FBP. Moreover, it was shown that the proposed method was able to reconstruct projection data acquired from various CT scanning geometries such as fan-beam and parallel-beam (Figure 6).

Although the aforementioned RBF-NN and RNNs initially demonstrated promising results, they have been rapidly surpassed by CNNs. CNNs have the ability of capturing spatially oriented patterns in imaging data, which makes them particularly suited to reconstruct CT scans. An example of such a CNN approach was proposed by Würfl et al,^19^ who demonstrated that the traditional FBP method can be expressed in terms of CNNs. Their CNN consisted of a single convolutional layer to mimic the filtering of FBP, and a fully connected layer to learn the backprojection step. They found that the CNN achieved comparable results to traditional FBP while markedly reducing the computational complexity required to perform the reconstruction. In addition, they showed that their framework can be extended to mimic the Feldkamp David and Kress (FDK) algorithm that is commonly used to reconstruct CT scans acquired with the cone-beam geometry (Figure 6).^26^

Another way of using CNNs for CT image reconstruction is to incorporate them within IR algorithms. For example, Adler and Öktem replaced forward operations of the iterative gradient descent reconstruction algorithm^20^ and the primal-dual hybrid gradient algorithm^21^ by partially trainable CNNs. In addition, various CNN approaches have been developed to improve reconstruction quality^34,36,37^ and computational efficiency^33^ of IR algorithms.

A different strategy was taken by Chen et al, who developed a CNN-based framework to find a direct mapping between CT projection data and reconstructed CT scans.^22^ Their framework used 50 iteration-inspired layers that each consisted of three learned convolutional operations. This framework significantly outperformed state-of-the-art reconstruction approaches. Moreover, they showed that this approach can be effectively used on incomplete projection data, which is a common problem in clinical practice, since radiation dose often needs to be reduced in order to comply with the ‘as low as reasonably achievable’ (ALARA) principle. The CNN-based IR framework was further improved by Xie et al,^41^ who implemented a learnable back-projection step that was previously fixed.

A different way of finding a mapping between projection data and reconstructed CT scans was developed by Fu and De Man.^28^ However, instead of finding a *direct* mapping, they proposed a hierarchical CNN in which the difficult reconstruction problem was split up into multiple intermediate steps that can be easily learned. This approach does not only improve the quality of reconstructed CT scans, but also gives clinicians an insight into the intermediate steps learned by the CNN. Similarly, Wang et al,^40^ developed two coupled CNNs to convert sinograms into CT images. The first CNN takes sinograms as input and converts them to data that are better suitable for the FBP or FDK algorithms. The output of the FBP or FDK algorithms (in the image domain) is subsequently fed to the second CNN, which further improves the reconstructed image quality. Ma et al^39^ also aimed to reconstruct CT images directly from the projection data. However, instead of breaking down the reconstruction challenge into multiple steps, they applied a combination of fully connected layers and convolutional layers to reduce the memory space requirement. They showed that their proposed approach results in substantially better image quality than standard FBP.

Ever since it was introduced in 2015, the U-Net has become a popular CNN architecture for medical image analysis.^85^ The U-Net consists of two convolutional paths. The downsampling path (*i.e.* encoder) creates a low-dimensional representation of the input data in order to capture local patterns. The upsampling path (*i.e.* decoder) subsequently captures global patterns through a series of upsampling steps. Both paths of the U-net are interconnected with skip-connections that allow the network to combine learned patterns at various scales. Applications of U-Nets for medical CT image reconstruction include the estimation of incomplete CT projection data (*e.g.* sparse-view and limited-angle CT)^27,37,38^ and improving IR, specifically the projected gradient descent reconstruction algorithm, by replacing the forward projector with a U-Net.^23^ Furthermore, variants of U-Net such as dual-frame and tight-frame U-Net have been proposed to reduce streaking and blurring artifacts in sparse-view CT scans during post-processing.^24^ Finally, U-Net has also been employed to predict deformation vector fields used to reconstruct 4DCT images.^35^

In summary, a wide variety of NN approaches have been proposed for CT image reconstruction (Table 1). In particular, CNNs have shown to be an incredibly interesting research topic with many new recent publications. Three CNN approaches were identified that are particularly interesting for the current maxillofacial CAS workflow. The first CNN approach aims to replace the computationally demanding forward operations within current IR methods.^20,21^ This markedly reduces the time constraint of applying IR methods in the maxillofacial CAS workflow. The second interesting CNN approach identified in this study is capable of reconstructing CT scans from incomplete projection data.^27,31,37^ Such approaches would enable clinicians to acquire high-quality CT scans of patients using low dose protocols. The third CNN approach replaces the total reconstruction process,^22,28^ which means that the CNN is trained to directly reconstruct CT scans from raw projection data. However, it must be noted that such fully learned reconstruction approaches are computationally expensive and require large amounts of annotated training data, which are not always available.

### Image segmentation

**Figure 7.**
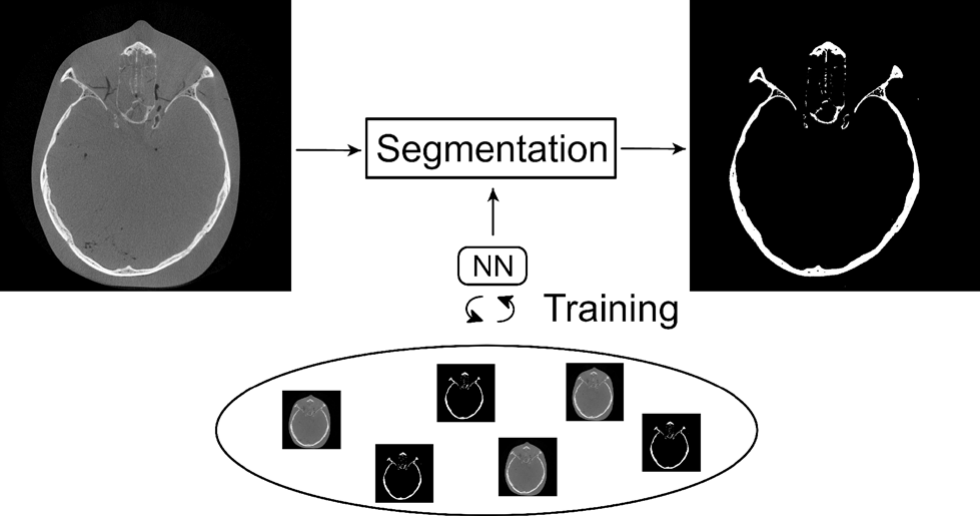
Schematic representation of the bone segmentation task required for maxillofacial CAS. A NN can be trained to automatically perform this segmentation task. CAS, computer-assisted surgery; NN, neural network.

Image segmentation refers to the task of labelling voxels of an image as a particular class (Figure 7). In the context of maxillofacial surgery, this image segmentation step is typically used to distinguish bony structures from soft tissues or air. Although a large variety of statistical methods have been developed for bone segmentation, they usually require the intervention of a medical professional in order to produce an accurate output, mainly due to the lack of reliable Hounsfield units and the limited signal-to-noise ratio of CBCT scans. It is therefore desirable to automate this task as far as possible, thereby relieving the medical professional from this labor-intensive and time-consuming task, while also increasing the accuracy and consistency of the segmentation results. We therefore reviewed the NNs used to automate CT bone segmentation tasks.

The first study describing the segmentation of bone in CT scans using NNs was published in 2002.^42^ In this study, a hierarchy of MLPs was trained on small patches of head and chest CT scans. The trained MLPs subsequently classified the center pixels of the patches and combined all separate pixel classifications to create a segmented image. Although similar MLPs were adopted by Gassman et al^43^ (2008) and Kuo et al^44^ (2013) to segment the phalanges and the nasal septum, respectively, different input data were used to train the MLPs. Namely, Gassman et al provided spherical co-ordinates, probabilities and intensities of individual pixels as input for their MLP, whilst Kuo et al used single rows of CT scans.

Segmentation using NNs took a significant leap after the ground-breaking performance of a novel CNN architecture (AlexNet) developed by Krizhevsky et al. in 2012.^86^ Similar to the aforementioned MLPs, this CNN architecture was trained using a patch-based approach in which the CNN aimed to classify the center voxels of small image patches. Inspired by the performance of this patch-based CNN, researchers have shown that such CNNs can achieve similar and in some cases superior performances compared to state-of-the art statistical methods when segmenting the mandible,^46,52^ the spine^51,61^ and the skull^50^ in CT scans. Nevertheless, the clinical application of patch-based CNNs for segmentation has been limited since many redundant convolution operations are necessary to classify all image voxels, which significantly slows down training and increases segmentation times.

In order to overcome this challenge, Ronneberger et al published a variation of the traditional CNN architecture, known as U-Net.^85^ U-Net can directly provide a segmented image as output (*i.e.* semantic segmentation) which increases its computational efficiency compared to patch-based CNNs. As a result, this U-Net architecture has ever since been applied for several CT bone segmentation tasks. For example, Klein et al,^48,56^ and Noguchi et al,^67^ applied U-net to segment bone in whole-body CT scans and reported that the U-Net performed significantly better than the standard segmentation procedure, *i.e.* global thresholding combined with morphological operations. Furthermore, U-Net was used to segment vertebrae,^54,59,68,71^ teeth,^55,72^ pelvic bones,^62^ orbital bones,^57^ cochleae^69^ and cranial bones.^70^ In a different study,^45^ a CNN architecture very similar to the U-Net, namely SegNet,^87^ was used to perform edge detection and multiscale segmentation of the femur in CT scans. Finally, Lessmann et al extended the standard U-Net architecture in order to both segment and identify an a priori unknown number of vertebrae.^49,58^ Their proposed architecture was able to automatically identify the individual vertebrae, whilst having comparable segmentation performance as the standard U-Net.

Since high segmentation performances have been achieved by U-net across a large number of studies, U-Net is currently considered the state-of-the-art for CT bone segmentation. Nevertheless, alternative CNN architectures for medical image segmentation have also been widely employed in the reviewed papers. For example, Men et al proposed a deep dilated CNN (DDCNN), in which the first and last layers perform dilated convolutions in order to extract multiscale features.^47^ Such a dilated convolution refers to the inflation of a convolution kernel while leaving sparse spaces between its elements. This dilation thus increases the receptive field of the kernel without increasing the number of model parameters. Torosdagli et al^60^ segmented the mandible using a fully convolutional DenseNet, which is comparable to U-Net, but uses dense blocks instead of regular convolutional layers. These dense blocks comprise multiple densely connected convolutional layers, which means that each convolutional layer within the dense block is connected to all other layers in the block. Similarly, a UDS-Net^64^ has been proposed, consisting of a U-Net with a dense block and spatial dropout, to segment teeth. Furthermore, 3D-DSD net has been developed which consists of a U-Net with a dense block and additional skip connections.^65^ The use of dilated convolutions and dense connections was further exploited in the mixed-scale dense CNN (MS-D network),^88^ which was used to segment the mandible in cone-beam CT scans.^4^ In an MS-D network, each convolutional layer performs a dilated convolution and is densely connected to all other layers of the network. It was found that these properties allow the MS-D network to achieve comparable segmentation performances as U-Net, while using far fewer trainable parameters.^4^ Finally, Zhou et al developed the so-called N-Net, which is similar to U-Net but has an additional stream of downsampling layers,^53^ whereas Rao et al modified the U-Net by replacing the normal convolutions with so-called Deep Bottleneck Architectures.^73^

In this section, different NN approaches used for bone segmentation in CT scans were reviewed. Similar to the NN approaches used for CT image reconstruction, bone segmentation seems to be increasingly performed using CNNs in favor of alternative NNs. The CNN approaches identified in this review can be roughly categorized into patch-based approaches and semantic segmentation approaches. The patch-based approaches allow extracting a large number of patches from relatively few CT scans, which facilitates CNN training. Semantic segmentation approaches, on the other hand, annotate each voxel of an image during a single forward pass through the CNN, which is typically far more computationally efficient than patch-based approaches. As a result, current state-of-the-art CNN approaches usually perform semantic segmentation. Examples of such widely used CNN architectures are U-Net,^85^ ResNet^89^ and MS-D network.^88^

### Surgical planning

The final step in the maxillofacial CAS workflow is surgical planning. This step typically involves a combination of computer-simulated bone reconstruction and subsequent designing of appropriate patient-specific implants. For example, a simple method to reconstruct fractured bones in the skull is to mirror the bony structures from the contralateral healthy side.^90^ However, this technique is often constrained to small defects on one side of the skull. For larger defects, a complex procedure involving various 3D-modelling software packages is required. The success of NNs in image reconstruction and image segmentation calls for the application of similar techniques during surgical planning and implant design. In this section, we therefore review different NN approaches that have been applied for surgical planning.

An interesting area for automated surgical planning is the reconstruction of skull plates because of the simplicity of the local anatomical geometry. Already in 2001, efforts were made to automatically design skull implants.^74,75^ The authors of these papers used a single-layer MLP in order to reconstruct cranial bone from CT images of patients with skull defects. In order to optimize training of the MLP, an approach relying on orthogonal functions (Legendre polynomials) was employed. The presented MLP was able to significantly speed up and improve the design of skull implants. However, while mathematically interesting, this particular approach is unlikely to generalize to larger defects on the side of the skull, since Legendre polynomials are insufficient to correctly approximate such complex defects. A similar approach was proposed by Knopf and Al-Naji,^76^ which also approximates anatomical features using an MLP with a single layer of polynomial functions, specifically Bernstein polynomials. After being trained on a set of segmented CT slices, the network was able to reproduce the anatomical structure of healthy bone. The main advantage of this approach is that the output of the MLP is in the shape of curves that describe the bony structures, which can be easily loaded into CAD software. Nevertheless, it must be noted that most approaches based on curve fitting are currently restricted to the neurocranium where the geometry of the skull can be reasonably approximated by smooth curves.

A different way of employing NNs for surgical planning is to optimize parametrized implant designs. The advantage of working with parametrized designs is that the NNs do not require imaging data. In addition, since implant designs can often be described using a few parameters, relatively simple NN architectures can be used to optimize the parametrization of the design. This approach was, *e.g.* taken by Chanda et al,^80^ who optimized parameterized hip implants based on the effects of initial micromotion, stress shielding, and interface stress. Using a combination of a single-hidden-layer MLP, a genetic algorithm and finite element analysis (FEA), the authors deduced that the standard implant design can be significantly improved. A similar combination of an MLP, a genetic algorithm and FEA was also used in a different study in order to find the best combination of material properties and geometry to generate patient-specific dental molar implants.^81^ This approach was also used by Biswas et al,^82^ who optimized the design of patient-specific spine implants based on the bone condition, body weight and implant diameter.

Another well-attended problem in surgical planning is to predict how bone adapts to different loads. This bone adaptation can be caused by surgical implants and may lead to complications after surgery.^91^ To date, bone adaptation prediction has been commonly performed using FEA. However, researchers recently found that a combination of the well-established FEA method and an MLP can significantly speed up the computations.^77^ The inverse problem has also been studied, specifically the estimation of load parameters for a given bone porosity inferred from CT images. For example, Campoli et al^78^ showed how a single-layer MLP can solve this inverse problem and compute the load for a femur. The network was specific to a single femur and was trained using simulated data. After the introduction of noise to the training data, the network was still able to successfully estimate the load parameters for the femur.

An interesting NN approach that does not rely on the geometric properties of bone was employed by Morais et al.^92^ They trained a CNN, specifically a deep convolutional autoencoder, to reconstruct fractured or missing parts of the skull. This autoencoder learned a representation of a healthy skull and was able to subsequently reconstruct a portion of the skull that was artificially removed. However, the accuracy and applicability of this deep learning model to high-resolution data remains challenging due to the computing power necessary for training and validation. Moreover, the proposed method was only validated on MRI data and not yet on CT data.

A relatively new approach towards reconstructing tissue morphologies is to apply generative adversarial networks (GANs). GANs consist of two networks: an generative network for generating new, fictive images based on input images, and a discriminator for distinguishing the generated images from real images. In the context of morphology reconstruction, a GAN can be used to generate images of healthy tissues based on images of fractured or diseased tissues. The generated images can then be hardly, if not at all, distinguished from real images of healthy tissues. From a clinical perspective, such generated healthy images can be extremely useful as they provide a surgeon a view of what the result should resemble. Furthermore, generating healthy images can be particularly helpful when constructing patient-specific implants. An example of a GAN applied in such a setting can be found in the study by Liang et al,^84^ who developed a GAN to reconstruct the morphology of the mandible based on CT images of patients suffering from ameloblastoma or gingival cancer. Similarly, Kodym et al^83^ used a GAN to reconstruct the shape of defective skulls. Even though interesting approaches have been developed, relatively few studies have described NNs for surgical planning (Table 1). The majority of surgical planning studies included in this review implemented MLPs for applications such as dental implant design^80^ and prediction of bone adaptation.^77,78^ A possible explanation for the relatively few studies describing NN-based surgical planning might be that it is extremely difficult to develop a single network to account for all variations that clinicians face during surgical planning. For example, there are numerous possibilities of designing implants or surgical tools, and choosing an adequate design heavily depends on the available software tools on the market, the type of imaging data used, and the personal preferences of medical engineers. As a consequence, it is almost impossible to effectively train a NN to design implants if no constraints are imposed. Although a few studies solved this problem by using a parametrized implant design to reduce the degrees of freedom,^80,81^ this leads to more generic and less patient-specific implants. Hence, automating personalized surgical planning and implant design using NNs remains difficult. Nevertheless, the field is still currently active, as demonstrated by the recent AutoImplant Challenge^93^ that was created to motivate participants to develop automated methods for cranial implant design.

### Current challenges and future research

In order to further develop and validate NN approaches for the three main steps of the maxillofacial CAS workflow, challenges remain that need to be overcome. One of these challenges is the quantitative performance evaluation. To date, the SSIM and MSE have been commonly used to assess image quality in the CT image reconstruction step, whereas the DSC is commonly used to assess segmentation performances (Figure 4). These generic metrics, however, do not always represent clinical relevance. For example, maxillofacial surgeons often assess the surface of the bony structures in order to create a treatment plan and design implants. Therefore, surface-based performance metrics might be preferred over the generic metrics. One possible way of evaluating segmentation performance would be to convert segmented CT scans into virtual 3D models and subsequently calculating geometrical distances between a gold-standard virtual model and the NN-based virtual models.^94^ This approach enables the quantification of the surface quality of bony structures, and also allows the visualization and interpretation of the differences between the two virtual models.

Another well-known limitation of most NN approaches is the need for large amounts of paired training data, *i.e.* input and target.^95^ Although an extremely large number of medical images are acquired on a daily basis that can be used as input to train a NN, they commonly lack appropriate annotations. Annotating such medical images requires a high level of domain-specific expertise, in contrast to natural images that can be easily annotated through crowdsourcing. In order to avoid the challenge of acquiring annotated target images, an interesting research direction might be to develop semi- and unsupervised NN training approaches, which do not depend on annotated data sets to learn.

To date, the development of NN approaches has been mainly performed in academic settings. Although many different NNs have shown to improve the efficiency, accuracy and consistency in which clinical tasks can be performed, none of these NN approaches has, to the best of our knowledge, been approved for maxillofacial CAS by the United States Food & Drug Administration. As a consequence, the application of NNs in routine clinical tasks remains very limited. In order to allow for large-scale use of NNs in clinical settings, additional research is necessary that focus on the robustness of NNs when faced with large anatomical variations and different imaging characteristics. For example, two recent studies have already shown that a single CNN is able to accurately segment CT scans acquired using various CT scanners.^50,68^

The reconstruction of CT scans in the maxillofacial CAS workflow is typically optimized for visual interpretation by clinicians. This optimization is to be expected since visual assessment is often fundamental to establish correct diagnoses. However, a CT scan optimized for human interpretation might not be the ideal input for CNNs to perform the subsequent segmentation and surgical planning tasks. Namely, CNNs solely extract patterns from the CT scans without taking visual aspects into account. A possible way of improving the quality of the CAS workflow may therefore be to jointly train two CNNs to simultaneously reconstruct and segment a CT scan. Such joint training approaches have already shown promising results in lung nodule detection,^96^ and may open up promising avenues when used in the maxillofacial CAS workflow.

One of the limitations that was encountered in the reviewed publications is that the heterogeneity of the data used to train the NNs makes it particularly challenging to draw generic conclusions. NN models have been trained on CT images of various different anatomical regions or phantoms that have been acquired using a plethora of different scanners, or through simulation. In order to validate a methodology, benchmark data sets should be developed which would enable a better comparison between the NN approaches. Furthermore, any work performed on simulated data and/or phantoms should be validated on clinical data, as it is challenging to assess the clinical applicability of NN approaches that have been solely validated on synthetic data.

Finally, it must be noted that research on artificial intelligence, and deep learning in particular, is evolving at an incredible rate due to the unprecedented interest and resource investment these fields. As a consequence, the state-of-the-art on this topic will naturally evolve rapidly. Nevertheless, this does not mean that a review cannot be useful. In fact, literature reviews may be arguably even more important when considering such rapidly evolving topics, as they help to identify bottlenecks, and to suggest new lines of research to advance the field.

## Conclusion

This scoping review describes different NN approaches used in the three main steps of the maxillofacial CAS workflow, *i.e.* CT image reconstruction, bone segmentation and surgical planning. In recent years, CNNs have rapidly become the most popular NN approach for CT image reconstruction and image segmentation, whereas MLPs remain the most common approach in surgical planning. Although CT image reconstruction and bone segmentation have been widely explored fields of research, additional research is required on the application of NNs for surgical planning. In order to reach the full potential of NNs for maxillofacial CAS, future research should focus on overcoming the challenges addressed in this review.
